# No increased rates of COVID-19 breakthrough infections in Altötting, a Bavarian district with a history of environmental PFOA contamination—results from a retrospective observational study

**DOI:** 10.1007/s15010-024-02230-z

**Published:** 2024-03-13

**Authors:** Andreas Beyerlein, Stefanie Heinze, Caroline Quartucci, Katharina Katz

**Affiliations:** 1 Bavarian Health and Food Safety Authority, Oberschleißheim, Germany; 2 Bayerisches Landesamt für Gesundheit und Lebensmittelsicherheit, Lazarettstraße 67, 80636 München, Germany; 3 Institute and Clinic for Occupational, Social and Environmental Medicine, University Hospital, LMU Munich, Munich, Germany

Dear Editor,

Perfluorooctanoic acid (PFOA) is a persistent chemical that is widely used in industrial production but is suspected to have a long-term detrimental impact on the health of exposed individuals, including impaired immune protection [1].

However, evidence on the association between PFOA exposure and COVID-19-related immune protection is scarce. While a modest but not statistically significant reduction in vaccine-induced antibodies against COVID-19 was reported for higher occupational PFOA exposure [2], higher serum PFOA concentrations were associated with an impaired immune response to SARS-CoV-2 infection but not to COVID-19 vaccination in another study [3].

Here, we explored whether there were increased rates of COVID-19 breakthrough infections in the Bavarian district of Altötting, where PFOA was used in industrial production until 2008 in a chemical park. This resulted in large-scale contamination of the environment in parts of the Altötting district and increased exposure of the local population to PFOA, although recent longitudinal analyses of blood samples collected in 2018 and 2022 within a human biomonitoring survey indicated that PFOA concentrations diminished over time after restoration of the drinking water supply [4].

We performed a retrospective secondary analysis of pseudonymized data on COVID-19 cases confirmed by real-time polymerase chain reaction (RT-PCR). As described in more detail elsewhere [5], these data were originally collected by the local Bavarian Public Health Departments according to the German Infection Protection Act (Infektionsschutzgesetz, IfSG) and transmitted to the Bavarian Health and Food Safety Authority on a daily basis, together with further demographic and infection-related information, including vaccination status, previous COVID-19 infections and COVID-19-related hospitalization and/or death. We extracted the data of all 6,279,393 Bavarian cases reported between 1 January 2021 and 31 December 2022 as of 4 January 2023. This time span covered the first 2 years after the introduction of publicly available COVID-19 vaccinations in Germany on 27 December 2020.

We considered COVID-19 cases as breakthrough infections if (a) at least two vaccinations were reported and the date of the second vaccination was at least 14 days prior to disease onset (or to the reporting date, if the date of disease onset was unknown) or (b) if at least one vaccination had been reported together with a previous COVID-19 infection, irrespectively of their time distance to the onset of the recent infection.

After excluding 3,490,100 cases (55.6%) with unknown immunization status, we compared the number of breakthrough infections among all reported COVID-19 infections between the district of Altötting and the remaining parts of Bavaria over time in 2021–2022.

Furthermore, we compared regional vaccination uptake rates based on data from the Robert-Koch-Institute. Given that these data cover vaccine uptake numbers per vaccination center but not per place of residence of the vaccinated person, we compared COVID-19 vaccination rates in the district of Altötting and its neighboring districts with those in the remaining Bavarian districts.

All analyses were performed using R 4.3.1 (https://cran.r-project.org/). The analysis code deposited at the time of publication is available at https://osf.io/rjs97/.

Vaccination uptake rates were comparable between the Altötting region and the remaining parts of Bavaria (Fig. 1, upper panel). Furthermore, the patterns of COVID-19 incidence were similar in the district of Altötting and whole Bavaria, although certain incidence peaks were somewhat more pronounced in Altötting (Fig. 1, middle panel). On average, the COVID-19 cases from Altötting were slightly older than were those from the rest of Bavaria (similar to the general population), while the distributions of sex were comparable (Table 1).

**Fig. 1:**
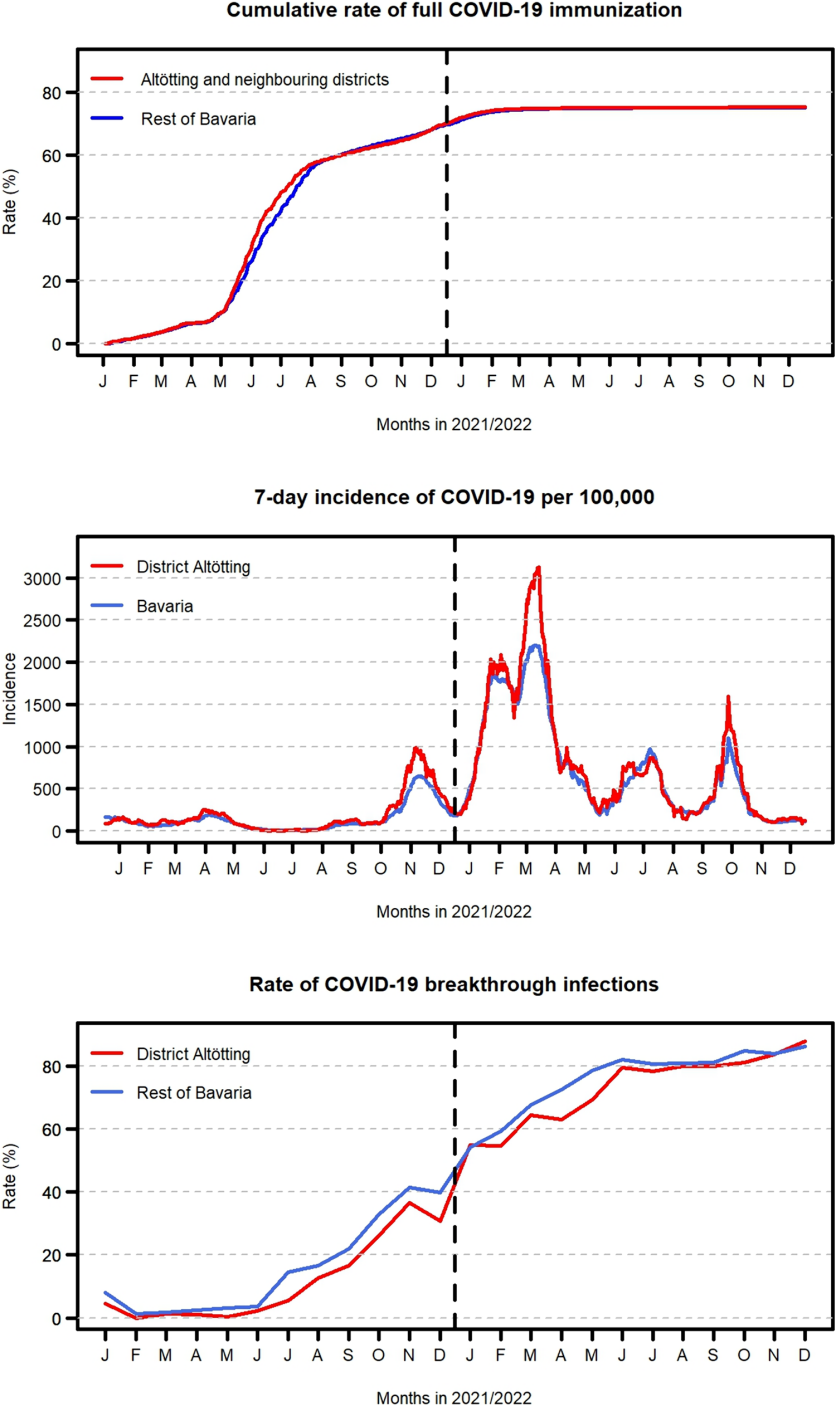
Cumulative rates of full COVID-19 immunization in the district Altötting and neighboring districts compared to the remaining parts of Bavaria (upper panel, based on data from the Robert-Koch-Institute), daily 7-day incidences of COVID-19 in the district of Altötting and whole Bavaria (middle panel), and monthly rates of breakthrough infections among all recorded COVID-19 cases with known immunization status in the district of Altötting and the rest of Bavaria (lower panel)

**Table 1:** Description of all 2,789,293 COVID-19 cases registered in Bavaria in 2021 and 2022 with known immunization status, stratified by their reporting district (Altötting or any other district in Bavaria)

	District Altötting	Rest of Bavaria
All cases	41,389 (100%)	2,747,904 (100%)
Sex		
Female	21,414 (51.7%)	1,426,976 (51.9%)
Male	19,853 (48.0%)	1,307,438 (47.6%)
Diverse	2 (0.0%)	292 (0.0%)
Unknown	120 (0.3%)	13,198 (0.5%)
Age		
0–19 years	8,623 (20.8%)	602,272 (21.9%)
20–34 years	8,942 (21.6%)	641,536 (23.3%)
35–59 years	16,307 (39.4%)	1,078,350 (39.2%)
60–79 years	5,720 (13.8%)	336,953 (12.3%)
80 + years	1,794 (4.3%)	88,210 (3.2%)
Unknown	3 (0.0%)	583 (0.0%)

All percentage values denote column percent

The rates of breakthrough infections increased over time but were slightly lower at most time points during 2021–2022 in the district of Altötting than in the rest of Bavaria (Fig. 1, lower panel). The age-stratified rates of breakthrough infections with a severe outcome (COVID-19-associated hospitalization and/or death) were similar in Altötting and the rest of Bavaria with 0.2% (33/18,671 cases with available information on immunization and severe outcome) compared to 0.2% (2998/1,775,219) in cases aged 0–59 years, 1.8% (62/3,537) compared to 2.3% (5908/254,037) in cases aged 60–79 years, and 13.2% (117/888) compared to 13.5% (7963/59,088) in cases aged 80 years or older, respectively.

Based on a large and timely surveillance dataset, these results do not indicate that COVID-19 breakthrough infections were more common or more severe in the Altötting district with its recent history of environmental PFOA exposure compared to the rest of Bavaria in 2021–2022. As expected, the number of breakthrough infections increased over time, along with increasing vaccination rates (and thus more vaccinated and fewer unvaccinated people available for infection) and also potentially due to the impact of waning immunization. As a potential limitation, the IfSG data contain no individual PFOA exposure measurements and cannot be linked with the human biomonitoring data for data protection reasons. We were therefore not able to match individuals with high PFOA exposure to those with low exposure and compare the two groups with respect to immune outcome, which would be another approach to investigate this research question. The immunization status and severity of the disease were unknown for a considerable number of COVID-19 cases, most likely due to exhausted capacities of the health authorities who collected the data. It should further be noted that our breakthrough infection rates refer to the number of COVID-19 infection records, not the number of vaccinated individuals.

## Data Availability

The individual infection data that support the findings of this study are not openly available due to reasons of sensitivity. The regional vaccination uptake rates collected by the Robert-Koch Institute (which were used as of 2 January 2023) are openly available under: https://github.com/robert-koch-institut/COVID-19-Impfungen_in_Deutschland.
